# Phase 1 Study of a Sulforaphane-Containing Broccoli Sprout Homogenate for Sickle Cell Disease

**DOI:** 10.1371/journal.pone.0152895

**Published:** 2016-04-12

**Authors:** Jennifer F. Doss, Jude C. Jonassaint, Melanie E. Garrett, Allison E. Ashley-Koch, Marilyn J. Telen, Jen-Tsan Chi

**Affiliations:** 1 Department of Molecular Genetics and Microbiology, Duke University, Durham, NC, United States of America; 2 Center for Genomic and Computational Biology, Duke University, Durham, NC, United States of America; 3 Division of Hematology, Department of Medicine, and Duke Comprehensive Sickle Cell Center, Duke University, Durham, NC, United States of America; 4 Center for Human Disease Modeling, Duke University, Durham, NC, United States of America; Cardiff University, UNITED KINGDOM

## Abstract

Sickle cell disease (SCD) is the most common inherited hemoglobinopathy worldwide. Our previous results indicate that the reduced oxidative stress capacity of sickle erythrocytes may be caused by decreased expression of NRF2 (Nuclear factor (erythroid-derived 2)-like 2), an oxidative stress regulator. We found that activation of NRF2 with sulforaphane (SFN) in erythroid progenitors significantly increased the expression of NRF2 targets *HMOX1*, *NQO1*, and *HBG1* (subunit of fetal hemoglobin) in a dose-dependent manner. Therefore, we hypothesized that NRF2 activation with SFN may offer therapeutic benefits for SCD patients by restoring oxidative capacity and increasing fetal hemoglobin concentration. To test this hypothesis, we performed a Phase 1, open-label, dose-escalation study of SFN, contained in a broccoli sprout homogenate (BSH) that naturally contains SFN, in adults with SCD. The primary and secondary study endpoints were safety and physiological response to NRF2 activation, respectively. We found that BSH was well tolerated, and the few adverse events that occurred during the trial were not likely related to BSH consumption. We observed an increase in the mean relative whole blood mRNA levels for the NRF2 target *HMOX1* (p = 0.02) on the last day of BSH treatment, compared to pre-treatment. We also observed a trend toward increased mean relative mRNA levels of the NRF2 target *HBG1* (p = 0.10) from baseline to end of treatment, but without significant changes in HbF protein. We conclude that BSH, in the provided doses, is safe in stable SCD patients and may induce changes in gene expression levels. We therefore propose investigation of more potent NRF2 inducers, which may elicit more robust physiological changes and offer clinical benefits to SCD patients.

***Trial Registration*:** ClinicalTrials.gov NCT01715480

## Introduction

Approximately 90,000–100,000 individuals in the United States are affected by sickle cell disease (SCD), with a higher incidence in Africa [1]. As the first described Mendelian disease [2], SCD results from a mutation of the sixth codon of β-globin that results in substitution of glutamic acid for valine. When deoxygenated, sickle hemoglobin polymerizes, altering red cell (erythrocyte) morphology, reducing cell deformability, and ultimately leading to hemolysis. Sickled red cells mechanically block blood flow in capillaries, while non-sickled red cells containing hemoglobin S aggregate, adhere to endothelial cells of post-capillary venules, and impair blood flow [3, 4]. Vaso-occlusion and defective oxygen delivery activate inflammatory pathways and lead to both acute symptoms and progressive organ damage in SCD [5, 6].

In addition to vaso-occlusion, hemolysis is another prominent feature of SCD that contributes to anemia [7, 8]. The freed hemoglobin from lysed erythrocytes sequesters critical antioxidants and accelerates an oxidative imbalance in vasculature that contributes to pulmonary artery hypertension and other pathological sequelae [9]. The increased hemolysis of sickle erythrocytes is caused in part by both higher levels of reactive oxygen species (ROS), and lower amounts of endogenous detoxifying antioxidants such as superoxide dismutase (SOD), catalase (CAT) and glutathione (GSH) [10–12]. The levels of these key antioxidants are regulated by NFE2L2, or NRF2 (Nuclear factor (erythroid-derived 2)-like 2). NRF2 is a master transcription factor that coordinates expression of oxidative stress capacity genes in response to physiological and pathophysiological oxidative stresses. Under baseline or unstressed conditions, NRF2 is sequestered in the cytoplasm by Kelch-like ECH-associated protein 1 (KEAP1) and targeted for proteasome-mediated degradation [13]. Under oxidative stress, NRF2 is stabilized, translocates to the nucleus, and binds to antioxidant response elements (AREs) within promoters of many antioxidant genes, including SOD, heme oxygenase-1 (HMOX1), and phase II detoxification enzymes such as NAD(P)H: quinone oxidoreductase (NQO1) [13, 14]. Interestingly, a previous global analysis of SCD erythrocyte microRNAs identified increased miR-144 levels were associated with increased disease severity; miR-144 downregulates NRF2, which results in lower NRF2 levels, and thus, contributes to SCD erythrocyte reduced oxidative stress capacity [15]. Additionally, NRF2 -/- mice develop hemolytic anemia, decreased erythrocyte GSH, and increased erythrocyte susceptibility to oxidative stress toxicity, which are also characteristics of SCD [16]. This evidence directly implicates NRF2 dysregulation in the pathogenesis of SCD.

Currently, hydroxyurea (HU) is the only FDA-approved SCD drug. Its benefits appear to be, at least in part, due to its ability to induce fetal hemoglobin (HbF) [17, 18]. Increases in HbF inhibit erythrocyte sickling and are correlated with overall reduced disease severity [19]. Though HU reduces disease complications and prolongs lifespan, the treatment elicits variable responses in patients [20]. Therefore, there is an urgent and unmet need for novel SCD therapeutics. A recent study demonstrated that NRF2 activation directly induced γ–globin transcription and increased HbF *in vitro* [21]. Accordingly, NRF2 activation may benefit SCD patients by both increasing HbF levels and enhancing oxidative stress capacity. The clinical benefits and therapeutic potential of NRF2 activation has been explored in several areas such as chemoprevention of prostate cancer [22], immunological disorders [23], and chronic kidney disease [24]. However, the therapeutic potential for NRF2 activation in SCD had yet to be investigated. Therefore, we proposed to activate NRF2 in SCD patients with sulforaphane (SFN), a well-known, natural isothiocyanate enriched in cruciferous vegetables such as broccoli sprouts [25]. Broccoli sprout homogenate (BSH) is a natural, readily available product, which has been used as a NRF2 activator in several therapeutic areas [26–28].

We conducted an open-label, dose-escalation clinical trial to identify the safety and physiological effects of NRF2 activation, via BSH ingestion, in SCD patients. We hypothesized that activation of NRF2 in SCD patients with BSH would both enhance oxidative stress capacity and increase HbF levels in SCD erythrocytes. In this pilot trial, the primary objective was to determine the safety and tolerability of oral ingestion of BSH in SCD patients. The secondary objective was to identify whether ingestion of BSH could elicit physiological response markers indicative of NRF2 activation. We hypothesized that: (1) BSH can be safely tolerated in patients with SCD, and (2) BSH administered *in vivo* would alter parameters reflecting NRF2 activity.

## Materials and Methods

### Ethics Statement

The Duke University Institutional Review Board approved the study (Pro00033630), and all patients provided signed, informed consent. This study was registered with clinicaltrials.org (NCT01715480), and was performed using Good Clinical Practice. The trial protocol (S1 Fig) and checklist (S2 Fig) are found here:

### Patient recruitment

Adult patients were enrolled for this study at the Duke Comprehensive Sickle Cell Clinic. Patients were recruited based on the following criteria: diagnosis of hemoglobin HbSS or HbSß° thalassemia by electrophoresis, age ≥18 years, no evidence of ongoing symptomatic vaso-occlusion, hematocrit (Hct) ≥20% and hemoglobin (Hb) >6.0 g/dL, capacity to understand and sign informed consent, and ability to adhere to the daily regimen of BSH. Exclusion criteria included RBC transfusion or change in HU dose during the three months prior to study entry, ongoing pregnancy, diabetes, renal insufficiency (BUN >21 mg/dL and/or creatinine >1.4 mg/dL), or a history of allergy to sulfonamides.

### Study Design

**Fig 1:**
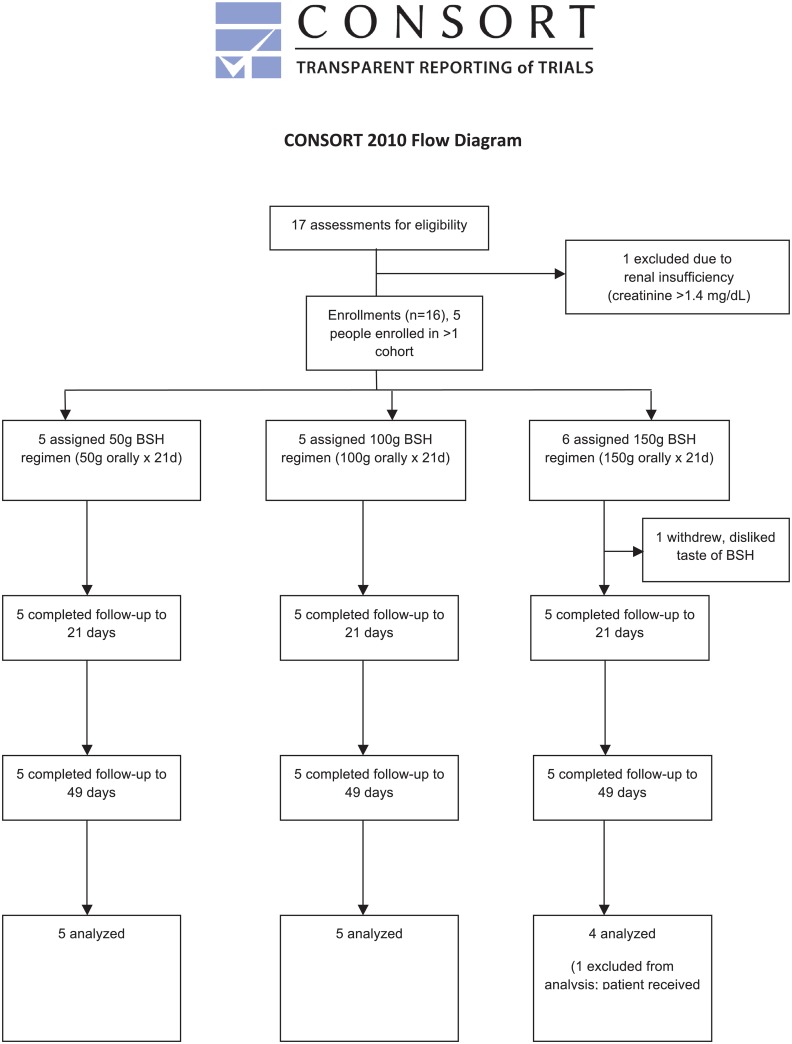
CONSORT flow diagram enrollment summary

The study design is outlined in Fig 1. Patients ingested a thawed preparation of BSH once daily for 21 days. Tolerability, toxicity, and physiological effects of NRF2 activation were determined based on comparisons between pre-treatment baseline (day 0), the last day of ingestion (end treatment, day 21, +/- 2 days), and after a washout period (day 49, +/- 7 days).

Patients were asked to avoid supplementary antioxidant vitamins, other cruciferous vegetables (such as broccoli, cabbage, and other vegetables in that family), as well as drinks with vitamin supplements at least two days prior to the pre-treatment visit, and until after the washout time point. We aimed to recruit five patients for each dose (50g, 100g, and 150g). The primary objective of this study was to evaluate the safety and tolerability of BSH in adult SCD patients.

After screening and the initial enrollment visit, which included giving the subject a week’s supply of study treatment, four follow up study visits were conducted for each subject in each cohort: week 1 (day 7), week 2 (day 14), week 3 (day 21, end of treatment), and week 7 (day 49, washout). At each visit, subjects were seen by a qualified health care provider, who obtained a medical history, including interim symptoms and healthcare utilization, concomitant medications, and transfusion. Other information collected at each visit included vital signs (height, weight, blood pressure, heart rate), pain score, and a focused interview that queried fatigue, review of BSH intake and ingestion of foods subjects had been asked to avoid, and symptoms/adverse events. Venipuncture was performed at each visit for CBC, LDH, blood chemistries, and, when scheduled, research labs. Urinalysis and CMP (complete metabolic profiling) were performed only for pre-treatment and end-of-treatment time points.

Safety and tolerability were determined by using data from adverse events, physical examinations, and clinical laboratory tests, including hemoglobin electrophoresis, reticulocyte count, CBC (complete blood count), LDH (lactate dehydrogenase) and serum chemistries (CMP). The secondary objective of this study was to identify blood biomarkers of NRF2 activation in SCD patients during the treatment period.

A Data and Safety Monitoring Board (DSMB) was established for monitoring this clinical study and consisted of three hematologists familiar with sickle cell disease. The DSMB met after each dose cohort was completed to review all safety data and to decide on approval for enrollment of patients at the next higher dose of BSH. The DSMB also determined the appropriate level of data and safety monitoring, reviewed reports of all serious adverse events (SAEs), and considered whether other actions (e.g. study closure, increased monitoring) were appropriate. All SAEs were also reported immediately to the IRB. Complications of SCD (e.g. pain episodes) were considered expected complications of SCD unless the DSMB judged their frequency to be overtly increased (which in no instance the DSMB did).

### Pharmacological Administration

Broccoli sprout homogenate (BSH) was prepared similarly to previously described methods [29]. Briefly, 50g of fresh Broccosprouts^®^ was homogenized with sterile water at a ratio of 1:1.2 (w/w) using a conventional blender. The mixture was frozen at -20°C in freezeable containers and patients were advised to add the mixture to a fruit smoothie to mask the bitter BSH flavor. Also, patients were instructed to allow the BSH to thaw before ingestion and avoid heating the homogenate, as this may inhibit the bioavailability of the SFN compound [30]. Patients were asked to consume the homogenate at preferably the same time of day, once daily for 21 days. Patients consumed one (50g), two (100g) or three (150g) packages, depending on the dose.

### Cell culture

Mobilized peripheral blood CD34+ cells (AllCells) were grown in StemLine II media containing Glutamine (Sigma) with 20% BIT (StemCell Technologies) and 1% Penicillin/Streptomycin (Gibco). On Days 0–7, cells were supplemented with growth factors IL-3 at 20ng/mL (Invitrogen), stem cell factor at 50ng/mL (Invitrogen), and erythropoietin (Epo) at 3U/mL (Calbiochem). On days 8–12, cells were only supplemented with IL-3 and Epo. Cells were treated with SFN (Calbiochem) or tert-Butylhydroquinone (TBHQ) (Sigma) in DMSO.

### RNA isolation

Blood samples were drawn into PAXgene Blood RNA tubes (Qiagen), and RNA was purified from whole blood using the PAXgene Blood miRNA Kit (Qiagen) according to the manufacturer’s protocol. RNA from *in vitro* cultured erythroid progenitors was isolated using the miRVana miRNA isolation kit (Applied Biosystems), according to the manufacturer’s protocol. DNA was removed from RNA samples with DNase (DNA-*free*, Life Technologies).

### RNA quantification

A total of 500ng of total RNA was used for reverse transcription with SuperScript II following the manufacturer’s instructions (Invitrogen). The cDNA samples were used to assess RNA expression by qPCR with the Power SYBRGreen PCR mix (ABI) and primers specific for indicated mRNAs; all primers have been described previously [21]. The qPCR reactions were performed using the StepOnePlus system (ABI). GAPDH was used for normalization.

### Data analysis

Analysis of mRNA levels from cultured erythroid progenitor cells was performed using unpaired Student t-tests with the GraphPad Prism4 software package (GraphPad Software Inc., San Diego, CA). All analyses of human data from the clinical trial were performed using SAS version 9.4 (SAS Systems, Cary, NC): first, data from each of the three dose cohorts (50g, 100g, and 150g) were analyzed separately to determine effects of different BSH doses on clinical outcomes over the course of the clinical trial (pre-treatment, post-treatment, and wash-out). Second, to determine the overall effect of BSH, all subjects were grouped together, and some data from subjects who participated in more than one dose cohort were removed so that each subject was included only once. Because this analysis was exploratory in nature, subjects enrolled in multiple doses were removed in two ways: once so that data from the highest BSH dose was kept and once so that data from the lowest BSH dose was kept. Due to the modest sample size of each group, we opted to perform Friedman’s test, a non-parametric repeated measures test for differences between groups when the outcome is ordinal, but not necessarily normally distributed.

## Results

### Treatment of erythroid progenitors with SFN induces NRF2 target mRNA expression

**Fig 2:**
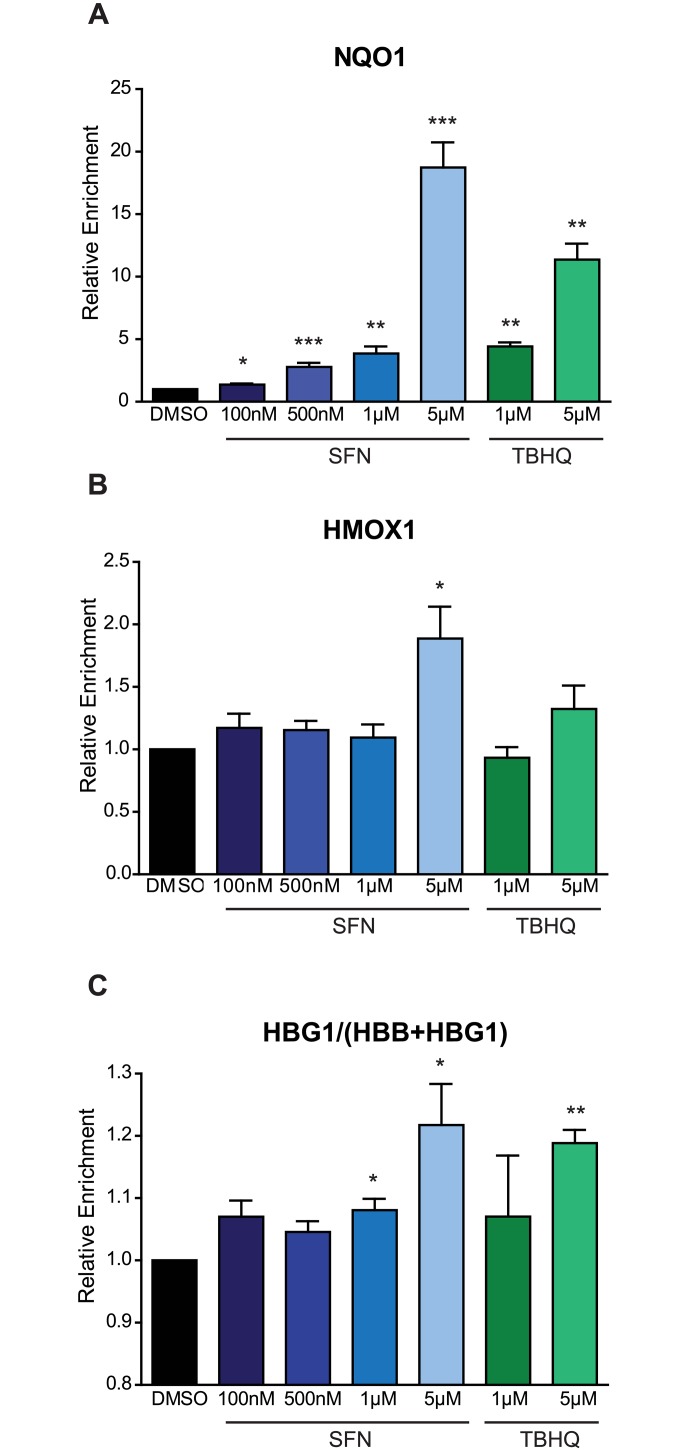
Treatment of erythroid progenitors with sulforaphane induces NRF2 target expression. Relative mRNA expression of *in vitro* differentiating erythroid progenitors 48 hours after beginning treatment, relative to untreated for A) NQO1, B) HMOX1, and C) HBG1/(HBB+HBG1) (n = 3). Cells were treated starting on Day 8 of differentiation, and were treated once every 24 hours, with a total of two treatments for the listed concentration. TBHQ was used as a positive control. All expression was set relative to pre-treatment values, was normalized with GAPDH, and all statistical analyses were performed using an unpaired t-test (* = p<0.05, ** = p<0.01, *** = p<0.001).

To determine whether SFN can activate NRF2 and induce NRF2-mediated transcriptional programs in erythroid cells, we treated differentiating human CD34+ erythroid progenitors with varying concentrations of SFN. TBHQ was used as a positive control, since it has previously been shown to increase NRF2 mRNA targets in erythroid progenitors [21]. Treatment of erythroid progenitors with SFN significantly increased the levels of two NRF2 target genes (*NQO1* and *HMOX1*) in a dose-dependent manner (Fig 2A and 2B). Next, we determined the ability of SFN to increase fetal hemoglobin levels through the expression of γ-globin (*HBG1*) relative to combined globin (β-globin + γ-globin, or *HGB1 + HBB*). At the highest SFN concentrations, we observed increased γ-globin mRNA relative to combined globin (γ/(β + γ)) after treatment (Fig 2C). As both γ-globin (a component of HbF) and β-globin (a component of HbA) form complexes with α-globin to constitute the hemoglobin tetramer (α2γ2, or α2β2, respectively), an increase in enrichment of γ–globin mRNA relative to β-globin mRNA is expected to increase total HbF levels. These results reflect the utility of SFN for NRF2 activation in erythroid cells, and provide a strong rationale for the therapeutic potential of SFN treatment in patients with SCD.

### Clinical Trial

We performed a phase I, open-label, dose-escalation clinical trial to determine the safety, tolerability, and efficacy of SFN-containing BSH in SCD patients. The study design is outlined in Fig 1. Subjects were required to ingest SFN-containing BSH over a prolonged period of time to allow for the replacement of circulating erythrocytes by cells affected by SFN during development. We aimed to enroll five patients at each dose if the smaller dose presented no safety concerns. Patients were enrolled for three dose levels of BSH: five patients at 50g, five patients at 100g, and six patients at 150g. Eventually, 11 subjects were enrolled and 10 were treated; five subjects participated in more than one dose cohort. There were a total of 16 enrollments. One subject, who participated in one dose cohort (100g), was diagnosed with HbSβ° thalassemia; all other patients were diagnosed with HbSS. One patient in the 150g cohort withdrew treatment and reported discontinuing only due to a dislike for the BSH flavor, but completed the end of treatment and end of washout visits for safety. Considering this partial treatment, we enrolled an additional patient into this cohort. However, this newly enrolled patient experienced a vaso-occlusive episode and received a blood transfusion before the end of treatment. Samples for the 150g cohort from the subject who did not complete treatment and the subject who received the transfusion were omitted from further analysis. Safety was assessed in both patients without any concerning events.

Only patients homozygous for sickle hemoglobin participated in the study. Demographic characteristics of the enrolled subjects are outlined in Table 1. In the 50g cohort, one patient was taking HU and four patients were not taking HU. In the 100g cohort, one patient was taking HU and four patients were not taking HU. In the analysed 150g cohort, two patients were taking HU, two patients were not taking HU, and two were excluded from analysis as described above.

**Table 1:** Demographic Characteristics.

	Dose		
	50g	100g	150g
Characteristic			
Statistic or Category			
*Age (years)*			
n	5	5	4
Mean	31.8	37.4	35.5
SD	5.5	9.3	11.2
Median	29	36	31.5
Min, max	28, 40	29, 52	27, 52
*Gender—n (%)*			
Male	60%	60%	25%
Female	40%	40%	75%
*Baseline Weight (kg)*			
Mean	63.8	64.7	72.9
SD	6	5.8	10.22
Median	66.7	65.3	69.25
Min, max	55.2, 69.8	55.4, 70.7	65.3, 87.6

### Subject safety

Adverse events (AEs) in this study were defined as unexpected medical occurrences or clinically significant abnormal laboratory changes occurring during the study, as according to the International Conference on Harmonization, Section E2A. Serious adverse events (SAEs) were those AEs that were life-threatening, or resulted in or extended hospitalization.

Changes in medical status from baseline were documented, although with few adverse events (Table 2). The adverse events were not thought to be related to BSH consumption. The most common adverse events were two instances of increased proteinuria for the same individual, and vaso-occlusive pain episodes experienced by two different individuals. Patients had a medical history of such episodes before starting the study. No remarkable trends in vital signs were observed during the study period.

**Table 2:** Adverse Events.

Type of adverse event	Number of events	Time period	Dose Cohort(s)	SAE?
Vomiting due to gastrointestinal infection	1	During treatment	50g	No
Hip pain	1	During treatment	50g	No
Vaso-occlusive pain episode with hospitalization	2	During treatment	50g, 100g	Yes
Shortness of breath with hospitalization	1	After treatment	100g	Yes
Increased proteinuria	2	During treatment	100g, 150g	No
Abdominal pain, vaso-occlusive pain episode, and QT prolongation, with hospitalization	1	During treatment	150g	Yes
Chest pain with hospitalization	1	After treatment	150g	Yes

All adverse events were not thought to be related to BSH consumption. Adverse events occurred as follows: one patient experienced both the vaso-occlusive pain episode (50g) and abdominal pain (150g) events. Another patient experienced the vaso-occlusive pain episode (100g), shortness of breath (100g), and chest pain (150g) episodes, and a different individual experienced both incidences of proteinuria (100g, 150g). All other events occurred within other, single patients.

Changes in clinical laboratory parameters were observed during the study period (Table 3). At the 150g BSH dose, post-treatment white blood cell (WBC) counts were lower (p = 0.0455) when compared to those obtained at pre-treatment. When examining all doses together and using the lowest dose for duplicate subjects, there was a statistically significant increase in platelets from pre- to post-treatment (p = 0.0082). This increase was not observed when examining all doses together and using the highest dose for duplicate subjects (p = 0.4795). While we observed trends towards increased platelets (50g), increased erythrocyte mean corpuscular volume (MCV, 100g), and decreased creatinine (150g), no changes were considered clinically significant. One complete blood count was missed during the study. Altogether, no serious safety concerns were observed after BSH ingestion for SCD patients.

**Table 3:** Clinical Parameters.

50g	Pre-Treatment	End Treatment	p-value
WBC (x10^9^)	9.1 (8.3–13.5)	9.4 (7.8–12.5)	0.9999
Reticulocyte (x10^9^)	485.9 (296.1–758.6)	350.0 (169.5–562.2)	0.1797
LDH (u/L)	405.8 (374–561)	404.0 (358–446)	0.6547
Creatinine (mg/dL)	0.64 (0.4–0.9)	0.68 (0.5–1.0)	0.3173
Platelet (x10^9^)	344.5 (195–456)	370.3 (226–507)	0.0833
RBC (x10^12^)	2.81 (2.43–3.60)	2.73 (2.06–3.71)	0.5637
Hbg (g/dL)	9.15 (8.2–10.2)	8.83 (7.4–10.5)	0.5637
BUN (mg/dL)	6.2 (3–9)	7.0 (4–13)	0.9999
Hct (%)	25.3 (22–29)	24.8 (22–30)	0.5637
MCV (fL)	90.3 (79–98)	91.8 (80–102)	0.3173
100g	Pre-Treatment	End Treatment	p-value
WBC (x10^9^)	10.3 (5.9–13.5)	10.8 (7.8–12.5)	0.6547
Reticulocyte (x10^9^)	390.0 (133.2–751.3)	450.3 (216.2–608.4)	0.1797
LDH (u/L)	405.0 (216–523)	366.2 (228–478)	0.6547
Creatinine (mg/dL)	0.64 (0.4–0.9)	0.70 (0.5–0.9)	0.1573
Platelet (x10^9^)	328.0 (237–403)	348.6 (235–446)	0.6547
RBC (x10^12^)	2.86 (2.43–3.60)	2.85 (2.06–3.71)	0.6547
Hbg (g/dL)	8.60 (7.9–9.3)	8.66 (7.6–10.1)	0.6547
BUN (mg/dL)	7.4 (3–14)	7.8 (4–17)	0.3173
Hct (%)	25.0 (22–26)	25.0 (22–28)	0.9999
MCV (fL)	91.2 (66–118)	91.8 (66–119)	0.0833
150g	Pre-Treatment	End Treatment	p-value
WBC (x10^9^)	8.8 (7.5–9.7)	7.6 (6.5–8.9)	0.0455
Reticulocyte (x10^9^)	331.5 (217.7–520.6)	285.2 (227.0–337.5)	0.1797
LDH (u/L)	345 (185–531)	416 (203–572)	0.3173
Creatinine (mg/dL)	0.73 (0.5–1.2)	0.65 (0.4–1.1)	0.0833
Platelet (x10^9^)	344.5 (246–409)	374.3 (286–439)	0.3173
RBC (x10^12^)	2.88 (2.28–4.04)	2.75 (2.10–3.85)	0.3173
Hbg (g/dL)	9.28 (7.9–10.2)	9.05 (8.5–9.7)	0.3173
BUN (mg/dL)	6.3 (3–8)	7.8 (4–12)	0.3173
Hct (%)	26.3 (22–30)	25.8 (24–29)	0.3173
MCV (fL)	95 (74–118)	96.2 (75–123)	0.3173

Average and range of clinical values for each cohort at each time point. Nominal p-value indicates difference between pre-treatment and end treatment using Friedman’s test.

### NRF2 mRNA targets are upregulated after treatment with BSH

**Fig 3:**
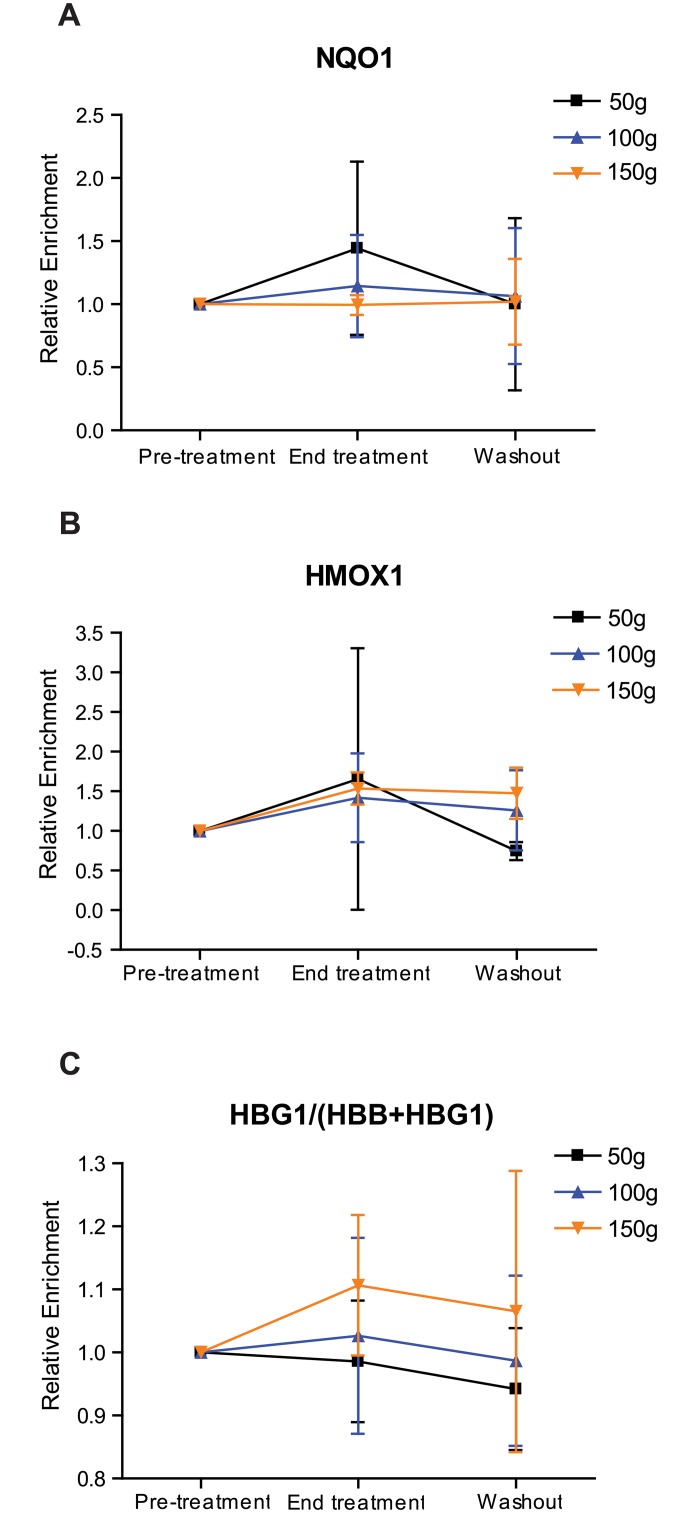
Effect of BSH ingestion on whole blood mRNA in SCD subjects. The composite of the mean for relative mRNA expression of A) NQO1, B) HMOX1, and C) HBG1/(HBB+HBG1) for all patients during the study period. All expression was set relative to pre-treatment values. Expression was normalized with GAPDH, and all statistical analyses were performed using Friedman’s test (* = p<0.05).

Next, we determined whether prolonged ingestion of BSH resulted in upregulation of NRF2 mRNA targets in sickle cell subjects. Using whole blood, we assessed the relative levels of NRF2 target mRNAs across all cohorts during the study period (Fig 3). For those taking the 150g BSH dose, *hmox1* was significantly upregulated from pre- to post-treatment (p = 0.0455). Likewise, when doses were grouped together and the highest dose was retained for duplicate subjects, *hmox1* was significantly upregulated from pre- to post-treatment (p = 0.0196, data not shown). For those taking the 100g BSH dose, *hbg1* was also significantly upregulated from pre- to post-treatment (p = 0.0253). We also observed a trend toward increased mean relative for *hbg1* when doses were grouped together and the highest dose was retained for duplicate subjects (p = 0.10). Percent changes for all NRF2-induced mRNAs interrogated are shown in Table 4.

**Table 4:** Average percent change of mRNAs at end treatment relative to pre-treatment.

mRNA	BSH Dose		
	50g	100g	150g
*hmox1*	+65.5%	+41.8%	+53.5%*
*nqo1*	+44.2%	+14.4%	-0.7%
*hbg1*	+65.2%	+130.8%*	+21.6%
*hbg1*/(*hbb*+*hbg1*)	-1.4%	+2.6%	+10.7%

*significant difference between pre-treatment and end treatment (p <0.05)

Although most of these differences in target mRNA expression are not statistically significant, together they suggest that BSH ingestion led to a consistent, detectable trend of induction of NRF2-dependent gene expression in human blood cells.

### BSH treatment did not consistently induce HbF protein

**Fig 4:**
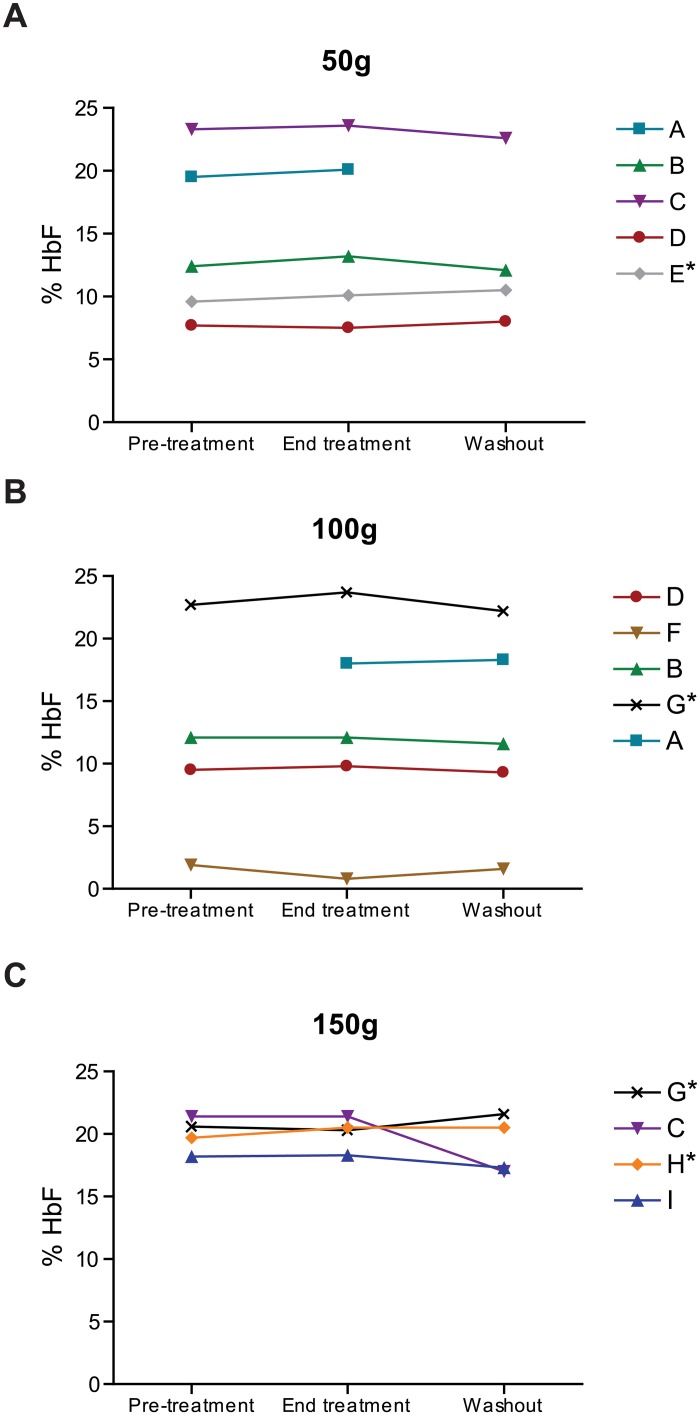
Fetal hemoglobin levels during study period. Fetal hemoglobin levels were assessed with hemoglobin electrophoresis for the A) 50g, B) 100g, and C) 150g cohorts. Each individual patient is designated with letter, symbol, and color codes; several patients participated in multiple cohorts. Patient F was diagnosed with HbSß° thalassemia; all other patients were diagnosed with HbSS. Patients taking hydroxyurea during the study period are denoted with an asterisk.

Finally, since we found that treatment of erythroid progenitors with SFN *in vitro* resulted in *γ-globin* upregulation (Fig 2C), and ingestion of BSH resulted in *γ-globin* upregulation (Table 4), we also assessed whether BSH consumption increased the relative levels of HbF protein in SCD subjects. Among all subjects who participated in and completed the trial, eight patients showed increases in HbF at end of treatment versus pre-treatment, two showed unchanged levels, and four patients experienced decreases in HbF. To clarify the potential confounding factor of HU usage, patients taking HU are indicated (Fig 4). In the 50g cohort, there was a modest but not statistically significant increase of average HbF from 14.5% (pre-treatment) to 14.9% (end treatment) (p = 0.1797) in the five subjects (Fig 4); a modest increase in HbF was observed for four of the five subjects. Interestingly, the only individual showing a decrease in HbF also presented the lowest baseline HbF values and had received a transfusion between three to four months prior to starting the study. This patient displayed elevated HbA (adult hemoglobin or α_2_β_2_) values early in the study (pretreatment: 7.6%, end treatment: 2.4%, washout: 0%), which might have impacted overall HbF values and may have diminished potential effects of HbF induction. When subjects from each dose were combined and the lowest dose was retained for multiple subjects, a trend toward increased HbF levels from pre- to post-treatment was observed (p = 0.0956). Subjects in the 100g and 150g BSH dose groups did not show a significant increase in mean HbF levels from pre- to post-treatment. Two hemoglobin electrophoresis lab samplings were missed during the study period. Together, these data indicate that in each dose group, there was a modest, but not statistically significant increase in mean HbF values at the end of the BSH ingestion period, relative to baseline.

## Discussion

Here we describe a phase I, dose-escalation clinical trial to determine whether ingestion of SFN-containing BSH can induce NRF2 activity with the potential to ameliorate SCD severity. This proof-of-concept study is the first to provide *in vivo* preliminary data regarding NRF2 activators in SCD patients. In general, we found that the prolonged ingestion of the BSH presented no significant health concerns. Such ingestion resulted in upregulation of NRF2 mRNA targets by the last day of ingestion, reflecting successful NRF2 activation in SCD patients. While average HbF increased slightly after BSH ingestion in the three dose groups, these increases were not statistically significant. Therefore, more potent NRF2 activators and a longer treatment period may be required for more robust HbF induction and potential clinical benefits.

In this pilot trial, ingestion of BSH was shown to be safe and tolerable. Although some subjects experienced typical SCD complications, no significant number or severity of adverse events was reported. In a previous study, five of 60 healthy subjects who ingested BSH over three days experienced mild, temporary side effects such as nasal congestion and gastrointestinal problems [26]. With the exception of the gastrointestinal infection likely not associated with BSH ingestion in one subject (50g cohort), no other patients in this study presented such side effects. The side effects from the previous study may be attributable to higher doses (175g-200g BSH) or the addition of myrosinase to improve SFN bioavailability. Therefore, the safety and risks associated with BSH ingestion will need to be more extensively explored through further studies with more participants, and, perhaps, higher doses.

Effective activation of NRF2 with BSH has been used to treat airway inflammation and impaired innate viral response [26, 28]. Nevertheless, continuous induction of NRF2 using BSH is not a convenient approach. Subjects in our study reported a bitter taste, and one subject withdrew from the study due to dislike for the taste of the BSH. The distaste for BSH and inconvenience of regular ingestion may reduce patients’ compliance. Additionally, compliance may be lower with higher doses. We attempted but were unable to monitor compliance by detecting SFN metabolites in blood plasma after single-dose BSH consumption [31] due to technical difficulties. Therefore, patient compliance was recorded using food diaries and reports to physicians.

BSH ingestion led to few statistically significant changes in clinical laboratory parameters. Conversely, in the 150g cohort, we observed a significant decrease in WBCs, which could ameliorate SCD severity [32]. Additionally, in the 150g cohort, we observed a trend towards decreased serum creatinine levels, albeit not statistically significant (p = 0.083); this increase may suggest that SFN has a potential ability to improve renal function. Among the four analyzed individuals, three patients experienced a decrease in serum creatinine levels. Renal complications are common to SCD, and contribute to 16–18% of SCD mortality [33]. As NRF2 activation improves diabetic nephropathy [34, 35], we hypothesized that NRF2 activation might also improve renal function in SCD patients. A potent NRF2 activator, Bardoxolone Methyl (CDDO-Me), improved renal functions in phase II clinical trials for diabetic nephropathy [36]. However, in a subsequent expanded clinical trial, a higher rate of cardiovascular events was observed with CDDO-Me than with placebo [24, 36]. Our data suggest that BSH can be safely ingested by SCD patients to activate NRF2. We suggest clinical studies of other NRF2 inducers, either alone or in combination with other treatments, to elicit a safe, robust physiological response in SCD patients that may also improve renal function.

To monitor the efficacy of NRF2 activation *in vivo*, we identified gene expression changes associated with BSH ingestion and found a statistically significant induction of NRF2 mRNA targets in patients taking BSH. Among the assayed genes, *hmox1* was reproducibly induced both *in vitro* and *in vivo* after treatment. These results suggest that *hmox1* levels may be used to monitor both the presence and degree of NRF2 induction in SCD subjects. Interestingly, the overall levels of *nqo1* decreased with increased BSH dose. This result is unexpected, as we expect dose-dependent induction of all NRF2 targets. This result may reflect a saturation point for NRF2-mediated *nqo1* induction or presence of a negative feedback loop. Previous studies [21, 37, 38], and our own *in vitro* data (Fig 2) indicate that NRF2 activation induced γ-globin mRNA and HbF protein. We observed a trend, but not a statistically significant increase in HbF protein in SCD patients taking BSH. Considering the small sample sizes and relatively brief treatment period, all data should be treated as preliminary and interpreted with caution. The level of NRF2 activation by SFN in BSH may not have been sufficient to elicit this particular physiological change. Consistent with the Multicenter Study of Hydroxyurea, it is also likely that a subgroup of patients may not respond to HbF induction [17]. Variation in NRF2 pathway activities of SCD subjects may also contribute to variable treatment responses. Enrollment of more participants in NRF2 inducer clinical trials will be necessary to identify differential response.

Though NRF2 induction via SFN directly regulates transcription of targets such as *HMOX1*, *NQO1*, and *HBG1*, SFN also produces other important biological effects. For example, SFN inhibits proliferation of prostate cancer cells by eliciting epigenetic changes such as differential promoter methylation [39] and histone deacetylation inhibition [40]. An understanding of the additional effects of SFN and other NRF2 activators will be critical for achieving optimal therapeutic utility and identifying potential side effects. More potent NRF2 activators may be required to provide more robust therapeutic utility. Purified SFN has been safely used in many clinical trials for various human diseases, including autism and recurrence of prostate cancer [22, 41]. A Bardoxolone Methyl derivative, RTA 408, has shown promise as an anti-inflammatory agent, as well as a mitigator of radiation-induced impaired hematopoietic cell proliferation [42, 43]. Additionally, a combination of NRF2 inducers and statins has been proposed to additively increase fetal hemoglobin in erythroid cells, with encouraging preclinical results [37].

There are several limitations to the study design. For example, several subjects were enrolled in more than one cohort; this raises issues of sampling errors to enrich for the subjects without toxicity. Furthermore, the use of hydroxyurea may complicate the subjects’ HbF responses to the BSH. Therefore, we recognize that this study is preliminary in nature and, as such, we have not adjusted the significance level for the number of tests performed. All the p-values presented in this manuscript are nominal p-values.

In spite of these limitations and the small sample size in this study, we thought it important to fully explore the data from this clinical trial in an effort to generate hypotheses for future research. While our data reflect only modest changes in NRF2-mediated gene expression, they are highly encouraging and suggest that treatment with more potent NRF2 activators over an extended period may lead to more significant, favorable physiological responses in patients with sickle cell disease.

## Supporting Information

S1 Fig Trial protocol. (DOC)

S2 Fig CONSORT checklist. (DOC)
