# Supplementary material for: Phase 1 Study of a Sulforaphane-Containing Broccoli Sprout Homogenate for Sickle Cell Disease
Source: PLoS One. 2016 Apr 12;11(4):e0152895. doi: 10.1371/journal.pone.0152895 (PMC4829228; doi:10.1371/journal.pone.0152895)
Supplement: S1 Fig — (DOC) [file pone.0152895.s001.doc]

**Research Summary**

**Principal Investigator:** Jen-Tsan Ashley Chi, MD PhD

**Co-Investigator:** Marilyn J. Telen, MD

1. **Protocol Title** – Physiological effect of sulforaphane obtained from brocoli sprouts homogenates (BSH) on the HbF and anti-oxidative capacity of human sickle red blood cells (SS RBC)
2. **Purpose of the Study** –

Primary Hypothesis: The reduced capacity to defend oxidative stress accounts for the accelerated hemolysis of SS RBC, but the causes for this defect are incompletely understood. We have analyzed microRNA in SS RBC and found that high miR-144 in SS RBC represses the protein and activities of NRF2, the master regulator of anti-oxidative capacity. In addition, low NRF2 then fails to repress expression of miR-144, forming a negative regulatory loop that maintains the high miR-144/low NRF2 in SS RBC. This dysregulation of miR-144-NRF2 is a critical sickle cell disease (SCD) feature likely to explain the reduced oxidative capacity. We propose to use sulforaphane homogenized from the commercially available fresh broccoli sprouts certified by Brassica Protection Products LLC, branded BroccoSprouts®, to activate NRF2 to restore anti-oxidative capacity and induce gamma and epsilon chain of the ß-like hemoglobins, which possess anti-sickling properties.

Secondary hypothesis: BroccoSprouts® homogenates (BSH) can be safely used in patients with SCD and may present a safe and effective means to induce HbF and enhance anti-oxidative stress capacity in SS RBC.

No Intent to Treat:

The study intends to determine physiologic effects of BSH on SS RBC but does not intend to treat.

**Specific Aims:**


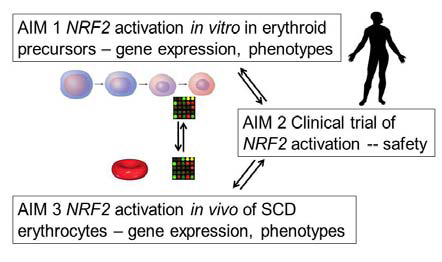


Aim 1: Examine the in vitro response of HbAA and HbSS erythroid cells to NRF2 activation by sulforaphane. We will focus on the expression of anti-oxidative genes and HbF. In addition, we will use microRNA arrays to identify NRF2-responisve microRNAs to enable the comparison with erythrocyte microRNAs in Aim 3, and monitor NRF2 activation in erythrocytes. We seek to enroll 3 volunteers with Hb AA (normal) and 3 SCD subjects with Hb SS disease to donate blood for this study. The subjects will not receive any compensation for their participation. The labs and procedures describe in the Aim 2 baseline study visit will be performed in this Aim 1 study.

Aim 2: Perform a dose-escalation pilot trial (procedures described #4 and #5 below) of sulforaphane. We will recruit cohorts of SCD patients to receive escalating doses of sulforaphane to determine its influence on symptoms and laboratory values indicating safety, erythropoiesis, hemolysis and HbF levels.

Aim 3: Analyze the changes in the microRNA expression and anti-stress phenotypes of purified SCD erythrocytes after sulforaphane treatment. These changes, after comparing with the microRNA changes after in vitro treatment (Aim 1), will be used to identify microRNAs indicating NRF2 activation. We will also determine whether high erythrocyte miR-144, given their association with severe defective anti-stress capacity, may benefit more from sulforaphane treatment.

1. **Background & Significance** –

Sickle cell disease (SCD) is one of the most prevalent genetic disorders in the United States (US), affecting more than 80,000 people. SCD is an inherited hematological disease. The most common form of SCD is caused by homozygosity for the hemoglobin S (HbS) form of the β globin gene on chromosome 11. SS RBC has a dramatic reduction in the anti-oxidative stress capacity. Compared to normal RBC, SS RBC are constantly exposed to an increased level of oxidative stress to have higher demand for antioxidant capacity caused by the auto-oxidation and instability of sickle hemoglobin, increased xanthine oxidase, higher level of leukocyte NADPH oxidase. These higher levels of oxidative stresses are likely to contribute to the hemolysis in SCD and resulting cascade of pathological mechanism associated with free hemoglobin and sequestration of vasoactive NO. However, SS RBC has a significantly lower anti-oxidative capacity. This reduced antioxidant capacity is an important pathogenic mechanism rendering SS RBC susceptible to oxidative insult and hemolysis. While such reduce oxidative stresses in SS RBC is well recognized, the basis for such defect is not known.

Recently, we have found that this reduced oxidative stress capacity in SS RBC is partially due to reduced levels of NRF2 (Nuclear factor-erythroid 2-related factor 2) repressed by high miR-144 in SS RBC. NRF2 is a basic leucine zipper transcription factor that is a master regulator of the anti-oxidant stress responses of most mammalian cells. Under unstressed conditions, NRF2 interacts with Keap1, which sequesters NRF2 in the cytoplasm and maintains basal expression of NRF2-regulated genes. Upon exposure to oxidative stresses and various electrophilic molecules, NRF2 is released from Keap1 and translocates to the nucleus, where it binds to the antioxidant response element (ARE) found in the promoters of key genes involved in oxidative stress responses. NRF2-regulated expression of ARE-driven genes, especially those involved in the biosynthesis and recycling of glutathione (GSH), involved in protection against oxidative stress, are critical for survival during oxidative stress in various in vivo and in vitro models. Deletion of NRF2 leads to hemolytic anemia in murine models, indicating its importance in erythrocytes. Therefore, we reason that activation of NRF2 may repair the defect in the oxidative stress capacity and improve outcomes of SCD.

Given the importance of oxidative stress capacity, many compounds have been developed to induce NRF2 and build the capacity to defend oxidative stresses in different disease setting. For example, sulforaphane [1-isothiocyanato-4-(methyl-sulfinyl)butane] is a natural dietary isothiocyanate isolated from broccoli and other vegetables. Sulforaphane is found to be a very potent inducer of phase 2 detoxication enzymes and other anti-oxidative capacity in cultured cells and animal models. Sulforaphane has been found to possess potent anti-carcinogenic activities and has been used as a chemo-preventive agent to forestall the development of cancers.

**Preliminary Studies**: Gene expression studies with sulforaphane exposure has demonstrated that transcriptional responses that are caused by NRF2 induction because of the covalent modification of Keap1 on its 27 cysteine residues, thus allowing NRF2 to translocate to the nucleus. Although NRF2 induction and sulforaphane have been used to boost anti-oxidative capacity and prevent many diseases, such an approach has not been applied to anemia disorders. This gap is especially surprising, given that oxidative stresses have been recognized as critical factors in the hemolysis of SCD and other anemia disorders. Based on our findings of miR-144-repression of NRF2 as a cause of compromised anti-oxidative capacity in SS RBC, we are proposing to test the potential of NRF2 induction to boost RBC anti-oxidative stress capacity and improve hemolysis and gene expression under the high level of stress seen in SCD. We believe that NRF2 induction should be regarded as distinct from anti-oxidant supplementation, since it ensures that the HbSS RBCs have enough enzyme capacity to survive stress environments instead of simply providing anti-oxidant substrates to HbSS cells that are already handicapped by insufficient anti-oxidant machinery. Furthermore, NRF2 activation also leads to the induction of HbF and epsilon forms of Hb known to inhibit the HbS polymerization and exhibit anti-sickling properties. Therefore, NRF2 activation may simultaneously have multiple benefits to reduce the hemolysis of SCD and improve clinical outcomes.

1. **Design & Procedures** –

Study Type: This is an open label dose-escalation study to test the efficacy of daily intake of sulforaphane via broccoli sprout homogenates (BSH) to achieve the physiologically significant but non-clinical endpoint of induction of HbF, glutathione and anti-oxidative capacity in SCD erythrocytes. Each patient will serve as his/her own control, as all endpoints will be measured at pre-treatment baseline, during treatment of BSH, and post-treatment washout.

Study Population: HbSS and HbSßº thalassemia

Primary Endpoint: Safety

**Preliminary sulforaphane level measurement revised procedure:**

Because our previous methods for processing blood for Sulforaphane (SFN) metabolite detection were not adequate. Four SFN metabolites: SFN-Cys, SFN-CG, SFN-NAC and SFN-GSH, should be detected in all the processed samples, yet only SFN-NAC was detected in all samples, albeit at levels so low that the amount of this metabolite could not be accurately identified with the usual standards. SFN-Cys, SFN-CG, and SFN-NAC could not be detected in any of the samples. This preliminary work is to allow optimization of the dosing preparation and the sample collection method to ensure that the amount of sulforaphane obtained from our preparation will be adequate.

We will test the ability to measure the increase of sulforaphane levels after ingestion of the non-toxic sulforaphane in a shake, smoothie, or unprocessed fresh or thawed out frozen broccoli sprouts in 20 subjects (10 subjects with sickle cell anemia and 10 healthy controls). To assess these serum levels, we must use a detailed protocol to stabilize the naturally unstable sulforaphane metabolite, and this sample must later be measured by our collaborators at the Oregon State University (Dr. Emily Ho). We must test the efficacy of our methods before proceeding with the main study to ensure that our detailed protocol is sufficient to stabilize the metabolite in the processed samples so that our collaborators at the Oregon Health can adequately test these metabolite levels.

Participants will be asked to donate three single blood samples by peripheral intravenous draw at about 30 minutes before ingestion, 4 hours after ingestion, and 6 hours after ingestion of 100, 150, or 200 grams of BroccoSprout® Homogenate (BSH). The maximum amount of blood that will be drawn is 10 ml at each time point for a maximum of 30 ml. Participants may be asked to repeat this procedure at different levels or preparation of BSH.

Secondary Endpoint: Physiological effects – HbF, GSH, anti-oxidative capacity and the expression of Phase II enzyme expression in SS RBC.

Projected Accrual: 50 patients for screening and 35 patients for participation in study.

COHORT 1 (5 patients) – Single 50 g BSH

↓

Review by DSMB. If Board approves,

↓

COHORT 2 (5 patients) – Single 100 g BSH

↓

Review by DSMB. If Board approves,

↓

COHORT 3 (5 patients)– Single 150 g BSH

↓

Review by DSMB. If Board approves,

↓

COHORT 4 (20 patients)– Single 200 g BSH

The 50 g BSH dosage may need to be adjusted to the minimal amount needed to have adequate Sulforaphane (SFN) metabolite detection.

If any serious adverse events occur, they will be reported immediately to the IRB. In addition, the study will be stopped until the DSMB reviews all available data and reaches a decision as to whether the study should be continued. The highest daily dose (200 mg) to be used in this study has been safely used in a previous trial. This dose will yield 102 μmol of sulforaphane. An escalating dose design is used in this study to monitor for safety in patients with sickle cell disease; also, previous studies show significant dose dependent increase on the response to BSH with greater response at the higher dose of 200 mg/day.

Study visits:

Screening/Baseline:

informed consent verification, medical history, review of concomitant medications, vital signs (VS), including height, weight, blood pressure, heart rate, pain, and fatigue. Venipuncture (up to 30 cc's) will be performed for CBC (complete blood count), LDH (marker for hemolysis), CMP (complete metabolic panel), baseline, hemoglobin electrophresis, HbF, Hb A2, SFN level, SFN-induced gene expression changes, RBC anti-oxidative capacity and gene expression.

Week 1 (Day 7): VS including height, weight, blood pressure, heart rate, pain, and fatigue, review of BSH intake (food diary) and concomitant medications, symptom/adverse event evaluation and continue food diaries; physical exam, vital signs (BP, pulse, temperature, height, weight, pain, and fatigue), review of concomitant medications. Venipuncture (up to 30 cc's) will be performed for CBC, LDH, SFN level. Review of food/fluids intake. Collection of sulforaphane supplement returned.

Week 2 (Day 14) VS including height, weight, blood pressure, heart rate, pain, and fatigue, review of BSH intake (food diary) and concomitant medications, symptom/adverse event evaluation and continue food diaries; physical exam, vital signs (BP, pulse, temperature, height, weight, pain, and fatigue), review of concomitant medications. Venipuncture (up to 30 cc's) will be performed for CBC, LDH, SFN level. Review of food/fluids intake. Collection of sulforaphane supplement returned.

Week 3 (Day 21): VS including height, weight, blood pressure, heart rate, pain, and fatigue, review of BSH intake (food diary) and concomitant medications, symptom/adverse event evaluation; medical history, review of concomitant medications, vital signs (VS), including height, weight, blood pressure, heart rate, pain, and fatigue. Venipuncture (up to 30 cc's) will be performed for CBC (complete blood count), LDH (marker for hemolysis), CMP (complete metabolic panel), baseline, HbF, SFN level, SFN-induced gene expression changes, RBC anti-oxidative capacity and gene expression.

Week 7 (Day 49, wash out): VS including height, weight, blood pressure, heart rate, pain, and fatigue, review of BSH intake (food diary) and concomitant medications, symptom/adverse event evaluation; medical history, review of concomitant medications, vital signs (VS), including height, weight, blood pressure, heart rate, pain, and fatigue. Venipuncture (up to 30 cc's) will be performed for CBC (complete blood count), LDH (marker for hemolysis), HbF, SFN level, SFN-induced gene expression changes, RBC anti-oxidative capacity and gene expression.

Participants while on the study will maintain a food diary. Each participant will receive instructions at the baseline visit on the use the food diary. The food diary data will be reviewed at each clinic visit.

The subjects who pass screening will receive sulforaphane in the form of broccoli sprout homogenates (BSH) for their intake at home daily for one week (plus 2 extra days’ supply). Subjects will return every week for the next 3 weeks of sequential visits.

1. **Selection of Subjects** –

*Inclusion Criteria*: Diagnosis of Hemoglobin (Hb) SS or Hb Sβ0 thalassemia by electrophoresis • Age ≥18 years • Hematocrit (Hct) ≥ 20% and Hb > 6.0 g/dL • Capacity to understand and sign informed consent and can adhere to the daily regimes of BSH

*Exclusion Criteria*: RBC transfusion during the 3 months prior to study entry • Pregnant• Currently receiving hydroxyurea treatments • Diabetes• Renal insufficiency (BUN >21 mg/dL and/or Creatinine >1.4 mg/dL) • History of allergy to sulfonamides

1. **Subject Recruitment and Compensation** – The consent process will occur in the Sickle Cell Center clinics at Duke South. All adult subjects may be approached for enrollment to the study if the inclusion criteria are met and they are patients. The PI or the research nurse will approach the potential participants. Participants will not receive any monetary compensation for their participation in the study.
2. **Consent Process** – Each subject must grant informed consent prior to enrolling in the study. No study procedure will be done prior to the patient consent. Participating subjects will be provided a copy of the dated and signed informed consent and the telephone numbers of the investigator and study personnel who can assist with any questions and or concerns.
3. **Subject’s Capacity to Give Legally Effective Consent** – Subjects will be 18 years, at of age or older and able to read and understand English.
4. **Study Interventions** – Prior to carrying out the procedure noted above in section #4, this study plans to recruit one subject to make sure that the methods for measuring blood serum Sulforaphane levels are adequate. We will test the ability to measure the increase of these levels after ingestion of the BSH shake for one student or employee. Nonparticipation or withdrawal from this study will not affect the student/employee's job status and nor grades. To assess these serum levels, a detailed protocol to stabilize the naturally unstable Sulforaphane metabolite will be used, and this sample will later be measured by our team of collaborators at the Oregon State University led by Dr. Emily Ho. We must test the efficacy of our methods before proceeding with the study to ensure that our protocol is sufficient to stabilize the metabolite in the processed samples so that our collaborators at the Oregon Health can adequately test these metabolite levels. The student will be asked to donate three single blood samples by peripheral intravenous draw at about 30 minutes before ingestion, 4 hours after ingestion, and 6 hours after ingestion of one thawed-out shake containing 50 grams of BSH currently stored at the DCRU.

**Risk/Benefit Assessment** – The investigators of the study do not expect the participants to receive medical benefit from their participation in this study. The tests provided may help participants learn about your general health. However, information from this study may help the investigators learn things about sulforaphane’s physiological effect on SS RBC that will help other patients with sickle cell disease in the future. This is a minimally invasive study that poses a low risk to the subject other than the general risk of loss of privacy and insertion of a venous catheter for blood draw. BSH at the doses proposed in this study have been safely used in many clinical trials for a variety of disease. Sulforaphane has a good safety profile and has been approved as broccoli sprout tea or homogenate for several human clinical trials.

1. **Costs to the Subject** – There will be no cost to subjects to participate in this study. The study will pay for the research-related items or services that are provided.
2. **Data Analysis & Statistical Considerations** –

Sample size: 50 patients for screening and 35 patients for participation. Patients will be allowed to participate in more than one dose cohort. This is a pilot study. The in vivo study in Aim 1 is designed for the participation of 6 subjects.

Primary Endpoint: Measurement of the HbF, anti-oxidative capacity, GSH and phase II enzymes and microRNA gene expression before and after the BSH injection.

Secondary Endpoints: Safety data regarding use of BSH in patients with SCD.

Descriptive statistics will be used to characterize changes from baseline in laboratory test results.

1. **Data & Safety Monitoring** – A DSMB will be established for monitoring this clinical study. DSMB members, appointed by Drs. Telen and Chi, will include at least three members with expertise in hematology or oncology. The DSMB will assess the risk of this clinical trial, determine the appropriate level of data and safety monitoring, review reports of all serious adverse events (SAEs), review the regularly submitted data and safety monitoring reports, and recommend appropriate actions (e.g. closure, increased monitoring) to the PIs.

All SAEs will be reported to the IRB, and the DSMB. The DSMB will review these data and notify the PI if safety appears to be an issue. If there are significant adverse events, the DSMB may recommend that the study be terminated. DSMB meetings for review of study data and decision of continuation will be scheduled as follows: after five patients complete the study at each dose level for cohort 1, 2, and 3; after twenty patients complete the study the 200 g dose level; or at any other times as deemed necessary by either the PI or any of the DSMB members.

1. **Privacy, Data Storage & Confidentiality** – The privacy rights of the subjects will be protected as required by law. All information that can identify an individual and the individual medical history will be kept confidential within the limits of the law. All study information will be kept locked in file cabinets accessible only by clinical research staff that are authorized and IRB-approved to participate in the study. Electronic information will be stored in secure electronic systems accessible only by Drs. Telen and Chi, and their clinical research team.

Table 1. Study Related Tests and Procedures

| Procedures | Screening Visit | Baseline (Day 0) | Day 7 | Day 14 | Day 21 | Day 49  (Wash out) |
| --- | --- | --- | --- | --- | --- | --- |
| Informed Consent | X |  |  |  |  |  |
| Clinic Visit | X | X |  |  | X | X |
| Interview to identify AE’s |  | X |  |  | X | X |
| Vital Signs | X | X |  |  | X | X |
| Physical Examination | X | X |  |  | X | X |
| MD screen, history | X | X |  |  | X | X |
| BSH dispensed |  | X | X | X |  |  |
| **Clinical Laboratory Tests** |  |  |  |  |  |  |
| Venipuncture | X | X |  |  | X | X |
| Complete Blood Count (CBC) | X | X |  |  | X | X |
| Reticulocyte count | X | X |  |  | X | X |
| LDH (marker of hemolysis) | X | X |  |  | X | X |
| Comprehensive Metabolic  Profiling (CMP) |  | X |  |  | X |  |
| Hemoglobin electrophoresis |  | X |  |  |  |  |
| HbF quant |  | X |  |  | X | X |
| Serum SFN level |  | X |  |  | X | X |
| **Research Studies** |  |  |  |  |  |  |
| Glutathione |  | X |  |  | X | X |
| Anti-stress capacity |  | X |  |  | X | X |
| Reactive Oxygen Species |  | X |  |  | X | X |
| RBC/Reticulocyte RNA |  | X |  |  | X | X |
| PAXgene whole blood |  | X |  |  | X | X |
